# Molecular mechanisms underlying *Nocardia* host interactions

**DOI:** 10.3389/fcimb.2026.1780562

**Published:** 2026-03-04

**Authors:** Bingqian Du, Ziyu Song, Min Yuan, Yuting Duan, Shuai Xu, Xiaotong Qiu, Zhenjun Li

**Affiliations:** 1 National Key Laboratory of Intelligent Tracking and Forecasting for Infectious Diseases, National Institute for Communicable Disease Control and Prevention, Chinese Center for Disease Control and Prevention, Beijing, China; 2 Wenzhou Key Laboratory of Sanitary Microbiology, Key Laboratory of Laboratory Medicine, Ministry of Education, China, School of Laboratory Medicine and Life Sciences, Wenzhou Medical University, Wenzhou, China; 3 Department of Epidemiology and Biostatistics, School of Public Health, Nanjing Medical University, Nanjing, China

**Keywords:** animal models, host interactions, molecular mechanisms, *Nocardia*, virulence factors

## Abstract

*Nocardia* species are opportunistic pathogens that cause localized and disseminated infections, particularly in immunocompromised individuals. Despite their clinical importance, the molecular mechanisms underlying *Nocardia* pathogenicity remain incompletely understood. This review summarizes current advances in *Nocardia* virulence factors, host immune responses, and intracellular survival strategies. A diverse array of virulence factors enables *Nocardia* to invade host cells, circumvent immune defenses, and maintain persistence within host tissues, including mammalian cell entry (Mce) proteins, antioxidant enzymes, phospholipase C, hemolysins, and siderophore-associated proteins. Host protection against *Nocardia* relies primarily on innate immune responses, with neutrophils playing a central role and being coordinated by γδT cells and interleukin-17–mediated signaling pathways. In addition, the clinical epidemiology of nocardiosis and animal models of *Nocardia* infection are also briefly summarized. However, most mechanistic studies remain restricted to a limited number of type strains. Further investigations into *Nocardia*–host interactions are essential for the development of improved diagnostic, therapeutic, and preventive strategies for nocardiosis.

## 1 Introduction

*Nocardia* spp. are intracellular, facultatively aerobic, Gram-positive actinomycetes that are widely distributed in the natural environment (Traxler et al., 2022). They are capable of infecting both humans and animals, and can cause severe infections, including pulmonary, cutaneous, and cerebral abscesses, with a worldwide distribution (Chen et al., 2017; Du et al., 2025a; Du et al., 2025b; Traxler et al., 2022). In recent years, the incidence and detection rates of nocardiosis have increased steadily, leading to a growing global disease burden. Notably, the all-cause mortality associated with *Nocardia* infections has been reported to be as high as 19.8%, and mortality among patients with central nervous system (CNS) nocardiosis is even higher, reaching 20.3%-30.5% (Du et al., 2025b; Shen et al., 2025). Nocardiosis is considered an opportunistic infection that primarily affects immunocompromised individuals, such as solid organ transplant recipients receiving immunosuppressive therapy, patients with low CD4^+^ T-lymphocyte counts, individuals with hematological malignancies, and those infected with human immunodeficiency virus (HIV) (Filice, 2005). Nevertheless, an increasing number of cases have also been documented in immunocompetent individuals, particularly among patients with bronchiectasis (Woodworth et al., 2017). The early diagnosis of nocardiosis remains challenging because of nonspecific clinical features, low bacterial burden, and difficulties in culture (Traxler et al., 2022; Filice, 2005). Therapeutic management is similarly difficult, often necessitating prolonged courses of antimicrobial therapy lasting several months or even years (Wilson, 2012; Song et al., 2025). Moreover, relapse is frequent, and the mortality rate remains high, especially among patients with underlying comorbidities (Coussement et al., 2016).

To date, most research on *Nocardia* has focused on clinical diagnosis and antimicrobial resistance, whereas the mechanisms governing host–pathogen interactions remain poorly understood. In this review, we systematically summarize and integrate current knowledge on the clinical epidemiology of nocardiosis, experimental animal models of infection, host–pathogen interaction mechanisms, and intracellular survival mechanisms of *Nocardia*. By synthesizing findings from clinical, microbiological, and immunological studies, this review aims to provide a more holistic understanding of *Nocardia* pathogenesis and to highlight key knowledge gaps that warrant further investigation.

## 2 Clinical features and epidemiology of nocardiosis

### 2.1 Clinical features

Nocardiosis presents in three major clinical forms: primary cutaneous infection, pulmonary infection, and disseminated infection. Uncommon sites of infection or clinical features include bacteremia, ocular infection, CNS involvement, mycetoma, and other extrapulmonary infections (Brown-Elliott et al., 2006). Primary cutaneous nocardiosis typically results from the direct inoculation of *Nocardia* into the skin following trauma, such as puncture wounds sustained during gardening with exposure to *Nocardia*-contaminated soil, traumatic injuries associated with motor vehicle accidents involving soil or dust contamination, or infections acquired through nosocomial exposure (Steinbrink et al., 2018; Tarchini and Ross, 2013; Welsh et al., 2011). It has been reported that approximately 80% of *Nocardia* species causing primary cutaneous infections are *N. brasiliensis* (Brown-Elliott et al., 2006). The lungs are the most frequently affected site, with pulmonary involvement observed in approximately 66.7% of reported cases. Primary pulmonary nocardiosis is generally attributed to the inhalation of aerosolized *Nocardia* organisms or mycelia and occurs predominantly in patients with chronic lung disease, individuals receiving corticosteroid therapy, and immunocompromised population (Du et al., 2025b; McNeil and Brown, 1994; Maggiorelli et al., 2015; Woodworth et al., 2017). Clinical features of pulmonary nocardiosis are often nonspecific and include cough, dyspnea, fever, and pleuritic chest pain, making early diagnosis particularly challenging (Traxler et al., 2022). Disseminated nocardiosis may develop secondary to either primary cutaneous or pulmonary infection, with subsequent hematogenous spread to noncontiguous organs or systems. Common sites of dissemination include the CNS, kidneys, joints, retina, and heart (Brown-Elliott et al., 2006; Beaman and Beaman, 1994; Shen et al., 2025).

### 2.2 Epidemiology

To date, no national surveillance or mandatory reporting system for *Nocardia* infections has been established, which precludes accurate estimation of the true incidence of nocardiosis. Available epidemiological data indicate that the estimated incidence ranges from approximately 0.33-0.87 cases per 100,000 population in Canada (Exmelin et al., 1996), 0.23-0.46 cases per 100,000 in the United States (Beaman et al., 1976), 0.45 cases per 100,000 in Spain (Yong et al., 2015), and 0.04 cases per 100,000 in Germany (Vuotto et al., 2011). Notably, the incidence increases dramatically among immunocompromised populations (Harpaz et al., 2016). Reported prevalence rates among heart transplant recipients range from approximately 0.65% to 2.5% (Santos et al., 2011; Peleg et al., 2007), and may reach as high as 13% in patients receiving azathioprine-based immunosuppressive therapy (Hofflin et al., 1987). Santos et al. reported prevalence rates of approximately 1.78% in lung transplant recipients, 0.26% in kidney transplant recipients, and 0.18% in liver transplant recipients (Santos et al., 2011). A national surveillance study in France further demonstrated that nocardiosis occurred at a rate of approximately 60 cases per 100,000 among patients with cancer, with an even higher incidence of approximately 701 cases per 100,000 among bone marrow transplant recipients (Torres et al., 2002).

Nocardiosis is relatively uncommon in individuals with HIV infection, which may be attributable to the routine use of trimethoprim–sulfamethoxazole prophylaxis for Pneumocystis Pneumonia; nevertheless, mortality among patients with HIV-associated nocardiosis remains high (Lederman and Crum, 2004; Du et al., 2025a). Moreover, although *Nocardia* species are distributed worldwide, substantial geographic variation in species prevalence has been observed (Du et al., 2025b). It should be emphasized that the high prevalence of *N. asteroides* reported in older literature should be interpreted with caution. Following the availability of molecular phylogenetic studies, strains previously typed as *N. asteroides* have been reclassified into distinct species, including *N. cyriacigeorgica*, *N. nova*, and *N. abscessus* (Roth et al., 2003). Therefore, many historical cases of *N. asteroides* likely represent these more accurately defined taxa. *N. nova* is the most frequently identified species in the United States (21.6-28%) (Hamdi et al., 2020; Yeoh et al., 2022) and Australia (29-35.5%) (Yeoh et al., 2022; Wei et al., 2021). In contrast, *N. farcinica* predominates in Belgium (44%) (Valdezate et al., 2017), China (29.1%) (Wang et al., 2023), South Africa (20.5%) (Lowman and Aithma, 2010), and France (20.2%) (Lebeaux et al., 2019), whereas *N. cyriacigeorgica* is most commonly reported in Iran (31.0%) (Hashemi-Shahraki et al., 2015), China (25.3%) (Wang et al., 2023), and Spain (25.3%) (Valdezate et al., 2017).

## 3 Animal models of *Nocardia* infection

### 3.1 Mice models

**Figure 1:**
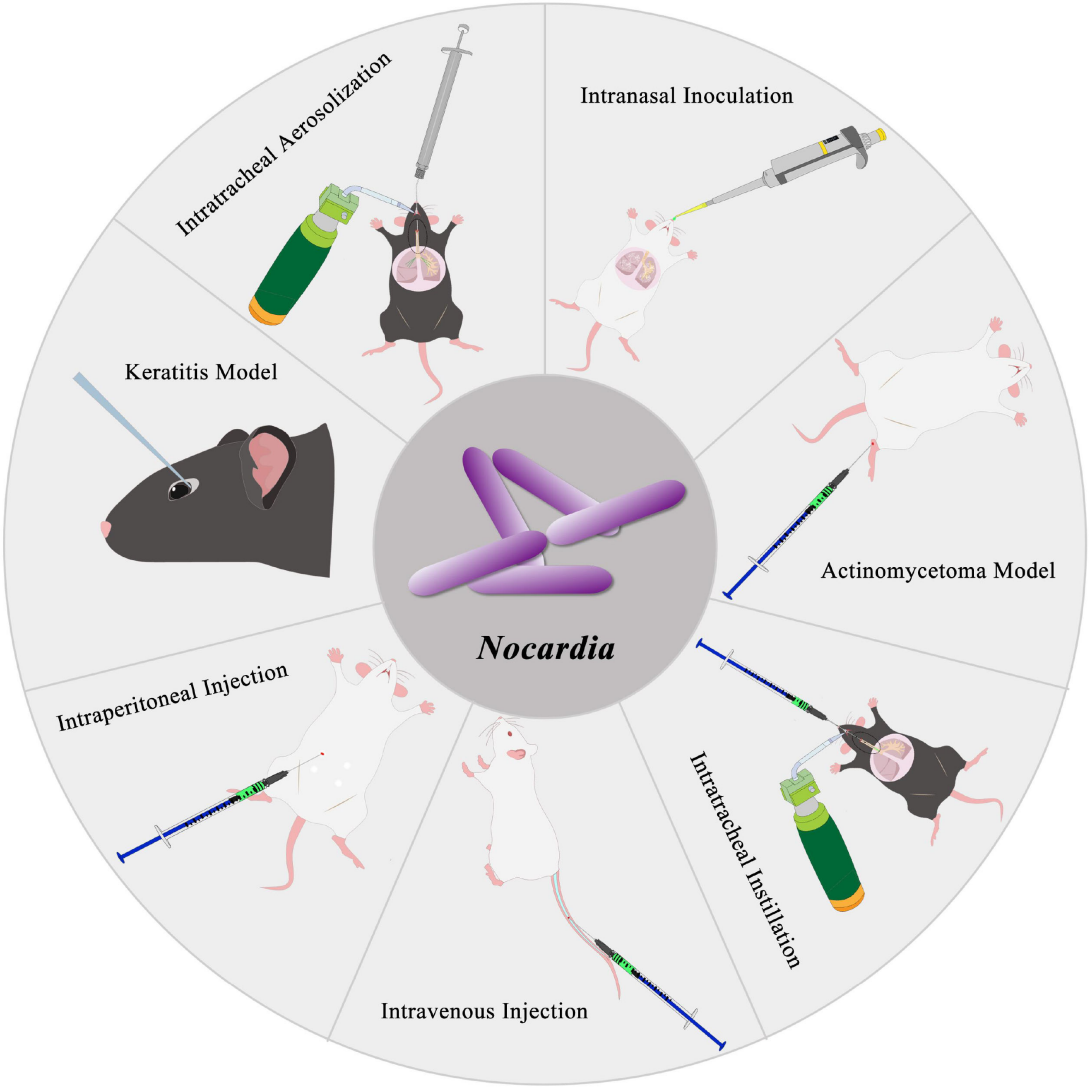
Schematic diagram of mouse models of *Nocardia* infection.

Animal models allow for reproducible assessment of disease progression and pathological changes, therefore it is indispensable for the development of novel therapeutics, vaccines, and diagnostic assays for nocardiosis. To date, well-established mice models have been developed for pulmonary nocardiosis, CNS *Nocardia* infection, *Nocardia* mycetoma, and *Nocardia* keratitis (**Figure 1**). As early as the 1960s, infection models of *N. asteroides* and *N. brasiliensis* were established in Swiss white mice (Folb et al., 1976; González-Ochoa, 1969). Subsequently, Salinas-Carmona et al. developed a BALB/c mice model of *N. brasiliensis*–induced mycetoma (Salinas-Carmona et al., 1999). Beaman et al. established a pulmonary nocardiosis model in Swiss Webster mice via intranasal inoculation (Beaman et al., 1978), which was further optimized by Mifuji Lira et al., who developed a granulomatous pulmonary nocardiosis model caused by *N. brasiliensis* in BALB/c mice (Mifuji Lira et al., 2016). Our laboratory established a BALB/c mice model of CNS *Nocardia* infection caused by *N. farcinica* and demonstrated that disease outcomes vary depending on the route of inoculation (Shen et al., 2024a). Both intravenous and intraperitoneal routes were shown to induce CNS manifestations in mice (Shen et al., 2024a; Ji et al., 2022). In addition, Guo et al. established a *Nocardia* keratitis model in C57BL/6N mice (Guo et al., 2024). More recently, our laboratory further optimized the pulmonary infection model by developing an intratracheal aerosolization model in C57BL/6J mice, which closely recapitulates human infection via inhalation of *Nocardia*-containing aerosol particles (Du et al., 2025c).

### 3.2 Other animal models

In addition to mice models, Sundararaj et al. established a guinea pig model of *N. asteroides* infection via intraperitoneal inoculation (Sundararaj and Agarwal, 1978). Mikami et al. developed a *Nocardia* infection model using the silkworm, which allows for quantitative evaluation of *Nocardia* pathogenicity as well as the therapeutic efficacy of antimicrobial agents against nocardiosis (Mikami et al., 2021). Furthermore, Bernardin Souibgui et al. established a nematode *Caenorhabditis elegans* model capable of detecting *Nocardia* strains involved in neurodegeneration, thereby markedly improving screening efficiency (Bernardin Souibgui et al., 2017).

## 4 Mechanism of *Nocardia-*host interactions

### 4.1 Virulence factors of *Nocardia*

**Figure 2:**
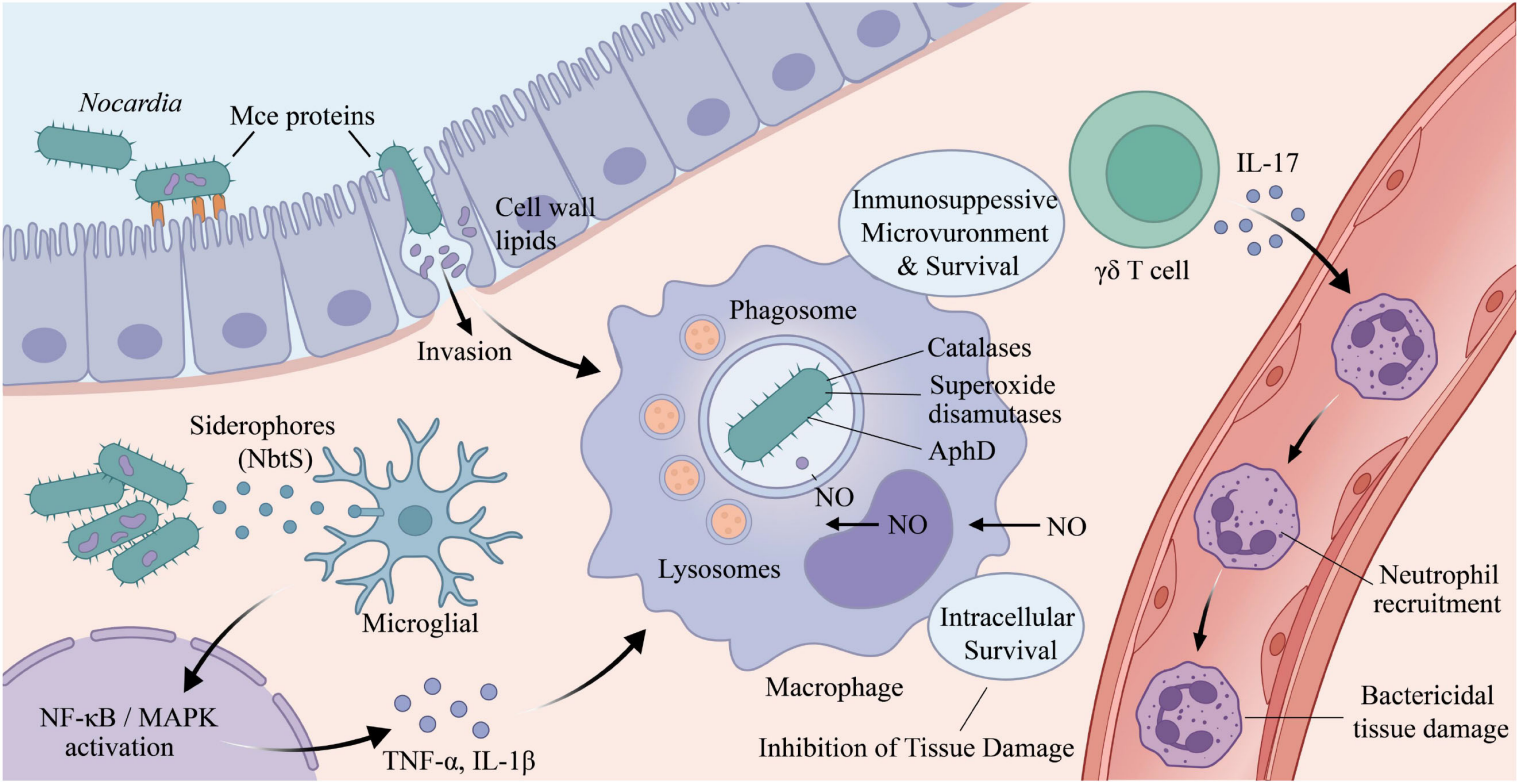
Mechanism of *Nocardia*-host interactions.

At present, studies on the mechanisms of *Nocardia* host interactions remain limited (**Figure 2**), and the pathogenic mechanisms of *Nocardia* are still not fully understood. Research on *Nocardia* virulence factors has primarily focused on type strains of *N. farcinica*, *N. brasiliensis*, and *N. cyriacigeorgica*, with virulence factors mainly comprising virulence proteins identified through animal and cell experiments, as well as putative virulence factors predicted by genomic analyses (Ishikawa et al., 2004; Zoropogui et al., 2013; Vera-Cabrera et al., 2013).

Invasion of host cells is a key pathogenic mechanism of intracellular pathogens. Mammalian cell entry (Mce) proteins are among the virulence factors of *Mycobacterium tuberculosis* and play important roles in host pathogen interactions (Arruda et al., 1993). Previous genomic sequencing studies have shown that *N. farcinica*, *N. brasiliensis*, and *N. cyriacigeorgica* each harbor six *mce* operons (*mce1* - *mce6*), which are involved in adhesion to and invasion of host cells (Ishikawa et al., 2004). In the absence of Mce proteins, *N. brasiliensis* completely loses its virulence in BALB/c mice (Gonzalez-Carrillo et al., 2016). Our previous studies demonstrated that the Mce1C and Mce1D proteins promote invasion of *N. farcinica* into HeLa cells and suppress host innate immune responses by modulating the NF-κB and MAPK signaling pathways (Ji et al., 2020b). In addition, *mce1E* may facilitate interactions between *N. farcinica* and mammalian cells (Ji et al., 2017).

Following host cell invasion, intracellular survival is a critical determinant of *Nocardia* pathogenicity. To counteract the oxidative burst generated by phagocytes, *Nocardia* expresses multiple antioxidant enzymes. Catalases degrade hydrogen peroxide, while superoxide dismutases degrade phagocyte-derived peroxides and singlet oxygen, thereby enabling Nocardia to persist and remain viable within phagocytic cells. Ishikawa et al. identified four catalases (*KatA*, *KatB*, *KatC*, and *KatG*), two superoxide dismutases (*SodC* and *SodF*), and one alkyl hydroperoxide reductase (AhpD) in *N. farcinica* IFM10152 (Ishikawa et al., 2004). Similarly, a catalase (*KatN*) and a superoxide dismutase (*SodS*) have been identified in *N. brasiliensis* (Vera-Cabrera et al., 2013). Collectively, these antioxidant enzymes are likely to play crucial roles in protecting *Nocardia* against phagocyte-derived reactive oxygen species and facilitating intracellular persistence.

Phospholipase C proteins may represent important virulence factors in *N. brasiliensis* but have not been identified in *N. farcinica* or *N. cyriacigeorgica* (Xia et al., 2017). Vera-Cabrera et al. identified four phospholipase C proteins (O3I_010265, O3I_012930, O3I_019520, and O3I_025065) in *N. brasiliensis* HUJEG-1 (Vera-Cabrera et al., 2013), suggesting a potential role for these enzymes in the pathogenesis of *N. brasiliensis*. However, direct evidence for their involvement in lung tissue damage or pulmonary dysfunction in nocardiosis remains lacking, as such effects have thus far been demonstrated only in other microorganisms (Wargo et al., 2011). Further functional studies are therefore required to clarify the contribution of phospholipase C to *Nocardia* virulence.

Hemolysins are recognized as important virulence proteins in bacterial pathogenesis (Adhikari et al., 2012). The genome of *N. brasiliensis* HUJEG-1 encodes four hemolysins (O3I_012605, O3I_013705, O3I_036360, and O3I_037730) (Vera-Cabrera et al., 2013), suggesting a potential contribution of these proteins to the pathogenicity of *N. brasiliensis*. Among them, O3I_012605 appears to be specific to *N. brasiliensis*, whereas homologs of the other three hemolysins can be identified in other *Nocardia* species. However, the precise roles of these hemolysins in *Nocardia* infection and host tissue damage remain unclear and require further experimental validation.

Siderophores are chelating compounds produced by pathogenic microorganisms to facilitate survival in iron-limited host environments. *Nocardia* species are capable of producing multiple siderophores, which have been implicated in host tissue damage during infection (Ikeda et al., 2005). Siderophore biosynthesis is associated with the *nbtA*-*H* gene cluster, which encodes two polyketide synthases (NbtB and NbtC), three nonribosomal peptide synthetases (NbtD, NbtE, and NbtF), two lysine-modifying proteins (NbtG and NbtH), and a receptor protein (NbtI) (Quadri et al., 1998). Functional studies have suggested a role for *nbtB* and *nbtS* in *N. farcinica* virulence, as deletion of these genes was associated with significantly improved survival in infected mice (Ji et al., 2022). Notably, the salicylate synthase NbtS has emerged as a pivotal mediator in the molecular pathogenesis of CNS nocardiosis. Beyond its metabolic role, *nbtS* has been implicated in triggering potent neuroinflammatory responses both *in vitro* and *in vivo*. Mechanistic investigations further demonstrated that NbtS directly interacts with microglial cells (BV2 and human microglial clone 3) and serves as a key ligand that activates the toll-like receptor 4 (TLR4)–dependent MyD88–IRAK4–IRAK1 signaling cascade. This engagement subsequently triggers the MAPK and nuclear factor kappa B (NF-κB) pathways, resulting in significantly enhanced pro-inflammatory responses, as evidenced by the massive production of tumor necrosis factor alpha (TNF-α) and interleukin-1β (IL-1β). Such excessive cytokine release is a critical driver of the neural tissue damage and high mortality associated with brain *Nocardia* infections. Collectively, these findings suggest that siderophore-associated proteins, particularly NbtS, are essential for *Nocardia*-induced neuroinflammation, though the full landscape of their role in systemic pathogenicity warrants further exploration (Shen et al., 2024b; Shen et al., 2025).

Genome sequencing analyses of *Nocardia* have indicated that its repertoire of putative virulence proteins includes invasins, nitrate reductases, proline–glutamate/proline–glutamate (PE/PPE) proteins, lipases, HBHA, NFA34810, and NFA52080. Some of these factors have been implicated in host infection, and previous studies have provided experimental evidence supporting the involvement of HBHA and NFA34810 in *Nocardia* virulence (Ji et al., 2020a; Beaman, 1996). However, the functional roles of many other predicted virulence-associated proteins remain largely unexplored and require further experimental validation.

Beyond protein-based factors, cell wall components play a regulatory role. Trevino-Villarreal et al. demonstrated that *N. brasiliensis* cell wall lipids modulate the responses of macrophages and dendritic cells, creating an environment that favors the development of experimental actinomycetoma (Trevino-Villarreal et al., 2012).

### 4.2 Host bactericidal mechanisms

Following *Nocardia* infection, the host rapidly initiates innate immune responses to eliminate both intracellular and extracellular pathogens. The essentiality of these defensive components is best illustrated by the heightened susceptibility of patients with specific immunodeficiencies, which allows for a systematic dissection of the host’s protective network. However, the immunological mechanisms underlying host defense against *Nocardia* remain incompletely understood. Clinical observations of fulminant infections in neutropenic patients or those with impaired neutrophil function further confirm that these cells are the primary executioners of bacterial clearance (Moore et al., 2000). Accumulating evidence indicates that neutrophils play a central role in host survival and the resolution of pulmonary nocardiosis. Shortly after intranasal inoculation with *N. cyriacigeorgica* GUH-2, bacteria invade the pulmonary epithelium and trigger a robust inflammatory response characterized by extensive neutrophil recruitment, ultimately leading to acute necrotizing pneumonia (Beaman, 1996). In the absence of neutrophils, *Nocardia* proliferates uncontrollably, potentially accompanied by enhanced invasion of pulmonary epithelial cells, resulting in increased cellular injury and aggravated histopathological damage (Moore et al., 2000). Consistently, blockade of CXCR2 signaling prior to infection significantly increases mortality in mice, further underscoring the indispensable role of neutrophil-mediated defense against *Nocardia* infection (Moore et al., 2000).

Beyond innate immune cells, γδT cells have emerged as key immunoregulatory lymphocytes involved in immune surveillance and maintenance of immune homeostasis (Hayday and Tigelaar, 2003). Increasing evidence suggests that γδT cells promote protective immunity largely through the induction and regulation of neutrophil responses (Hamada et al., 2008; Schulz et al., 2008; Shibata et al., 2007). In particular, interleukin-17 (IL-17) produced by γδT cells has been shown to induce CXC chemokines, granulocyte colony-stimulating factor (G-CSF), and adhesion molecules, thereby enhancing neutrophil recruitment, activation, and antimicrobial function against both intracellular and extracellular bacterial pathogens (Shibata et al., 2007; Kolls and Lindén, 2004; Lindén et al., 2005; Caccamo et al., 2011; Umemura et al., 2007; Markel et al., 2010). Notably, γδ T cell–deficient mice infected with a nonlethal dose of *N. cyriacigeorgica* GUH-2 develop severe disease and succumb within two weeks (King et al., 1999). Furthermore, Tam et al. demonstrated that γδT cells and IL-17 are essential for effective neutrophil infiltration and bacterial killing following *N. cyriacigeorgica* GUH-2 infection in mice (Tam et al., 2012). This experimental evidence mirrors the high prevalence of nocardiosis in patients with low CD4+ T-lymphocyte counts, such as HIV-infected individuals, highlighting that T cell orchestrated cytokine signaling is the mandatory “command center” for initiating an effective innate response (Filice, 2005). Collectively, these findings highlight a critical γδT cell–IL-17–neutrophil axis in host defense against *Nocardia*, although the precise regulatory mechanisms governing this immune network remain to be further elucidated.

While host bactericidal mechanisms aim to eliminate the pathogen, certain inflammatory mediators can paradoxically contribute to disease progression. Recent evidence suggests that nitric oxide (NO) may promote *Nocardia* pathogenesis rather than clearance. Salinas-Carmona et al. found that blocking inducible nitric oxide synthase (iNOS) protects mice from *N. brasiliensis*-induced actinomycetoma (Salinas-Carmona et al., 2020). Similarly, Yao et al. demonstrated that NO contributes to the pathogenesis of *N. farcinica* infection in both mouse models and alveolar MH-S macrophages (Yao et al., 2025).

### 4.3 Mechanisms of intracellular survival of *Nocardia*

Beyond adhesion to and invasion of host cells, the capacity of *Nocardia* to persist within host tissues and cells constitutes a critical pathogenic mechanism. Meester et al. reported that *Nocardia* infection induces macrophages and dendritic cells to differentiate into foam cells, thereby impairing their microbicidal functions, although the underlying mechanisms remain poorly defined (Meester et al., 2014). Beaman et al. further demonstrated that *Nocardia* can survive for prolonged periods within macrophages without being eliminated by phagocytic killing. This intracellular persistence is thought to result from multiple immune evasion strategies, including inhibition of phagosome–lysosome fusion, suppression of proteasomal activity, interference with phagosomal acidification, alteration of lysosomal enzyme activity, and resistance to oxidative killing. The critical importance of the host’s oxidative burst is underscored by the high clinical vulnerability of patients with chronic granulomatous disease (CGD), whose inability to generate reactive oxygen species (ROS) provides *Nocardia* a permissive environment for uncontrolled replication (Dorman et al., 2002). Collectively, these mechanisms compromise the bactericidal capacity of phagocytic cells and enable long-term intracellular survival of *Nocardia* (Beaman and Beaman, 1994; Barry and Beaman, 2007). These mechanisms are highly reminiscent of the intracellular survival strategies of *Mycobacterium*, which also manipulate the phagosomal environment to avoid lysosomal degradation. Furthermore, *Nocardia* can manipulate the host immune landscape to ensure persistence (Beaman and Beaman, 1994). Rosas-Taraco et al. reported that *N. brasiliensis* induces an immunosuppressive microenvironment characterized by specific cytokine profiles that that benefits its survival during the chronic stage of infection (Rosas-Taraco et al., 2012). Additionally, the pathogen directly impacts host cell viability; Navarro-Durán et al. demonstrated that *N. brasiliensis* infection induces macrophage cell death, a process that likely facilitates tissue damage and further dissemination of the bacteria (Navarro-Durán et al., 2022).

## 5 Current challenges and future perspectives

Despite significant progress in identifying the molecular determinants of *Nocardia* pathogenicity, several critical gaps remain. First, much of our current understanding is derived from a limited number of laboratory type strains, such as *N. farcinica* IFM 10152 and *N. cyriacigeorgica* GUH-2. Given the high genomic plasticity and clinical diversity of the *Nocardia* genus, it is unclear whether these mechanisms are universally conserved across emerging pathogenic species and highly resistant clinical isolates. Future research must prioritize the comparative analysis of clinical strains to capture the full spectrum of virulence.

Second, while genomic and bioinformatic tools have predicted a vast array of potential virulence factors, only a small fraction has been functionally validated. The integration of omics technologies, including transcriptomics, proteomics, and metabolomics, during *in vivo* infection is essential toward a dynamic understanding of host-pathogen interactions.

## 6 Conclusion

In conclusion, multiple virulence determinants, including Mce proteins, antioxidant enzymes, phospholipase C, hemolysins, and siderophore-associated proteins, collectively contribute to *Nocardia* host cell invasion, immune evasion, and tissue persistence. These virulence determinants not only facilitate tissue colonization but also enable evasion of host immune responses, particularly by interfering with phagocyte microbicidal functions, phagosome–lysosome fusion, proteasomal activity, and oxidative killing. Host defense against *Nocardia* relies heavily on innate immune mechanisms, with neutrophils and CXC chemokines forming a critical first line of defense. γδT cells and IL-17 further orchestrate protective neutrophil responses, highlighting a key γδT cell–IL-17–neutrophil axis in controlling infection. Despite these insights, the precise molecular mechanisms underlying *Nocardia* intracellular survival, immune evasion, and host–pathogen interactions remain incompletely understood. Future studies integrating genomics, functional assays, and animal models are essential to elucidate these pathways, which may inform the development of targeted therapeutics, vaccines, and diagnostic strategies for nocardiosis.
